# Does quantity matter to a stingless bee?

**DOI:** 10.1007/s10071-021-01581-6

**Published:** 2021-11-23

**Authors:** Johanna Eckert, Manuel Bohn, Johannes Spaethe

**Affiliations:** 1 Department of Comparative Cultural Psychology, Max Planck Institute for Evolutionary Anthropology, Deutscher Platz 6, 04103 Leipzig, Germany; 2 Department of Behavioral Physiology and Sociobiology, Biozentrum, University of Würzburg, Am Hubland, 97074 Würzburg, Germany

**Keywords:** Numerical cognition, Insects, *Trigona fuscipennis*, Associative learning, Quantity discrimination, Behavioral experiments

## Abstract

Quantitative information is omnipresent in the world and a wide range of species has been shown to use quantities to optimize their decisions. While most studies have focused on vertebrates, a growing body of research demonstrates that also insects such as honeybees possess basic quantitative abilities that might aid them in finding profitable flower patches. However, it remains unclear if for insects, quantity is a salient feature relative to other stimulus dimensions, or if it is only used as a “last resort” strategy in case other stimulus dimensions are inconclusive. Here, we tested the stingless bee *Trigona fuscipennis*, a species representative of a vastly understudied group of tropical pollinators, in a quantity discrimination task. In four experiments, we trained wild, free-flying bees on stimuli that depicted either one or four elements. Subsequently, bees were confronted with a choice between stimuli that matched the training stimulus either in terms of quantity or another stimulus dimension. We found that bees were able to discriminate between the two quantities, but performance differed depending on which quantity was rewarded. Furthermore, quantity was more salient than was shape. However, quantity did not measurably influence the bees' decisions when contrasted with color or surface area. Our results demonstrate that just as honeybees, small-brained stingless bees also possess basic quantitative abilities. Moreover, invertebrate pollinators seem to utilize quantity not only as "last resort" but as a salient stimulus dimension. Our study contributes to the growing body of knowledge on quantitative cognition in invertebrate species and adds to our understanding of the evolution of numerical cognition.

**Supplementary Information:**

The online version contains supplementary material available at 10.1007/s10071-021-01581-6.

## Introduction

Quantitative information is omnipresent in the world and plays an integral part in even the simplest tasks in our daily lives. We use quantitative information such as number, size, length, or weight to measure, rank, and order things. This kind of information allows us to understand and interact with the world in incredibly sophisticated ways. A subfield of quantitative cognition that has received much attention in the human literature is numerical cognition. Numerical cognition is different from other types of quantitative cognition because it focuses on discrete magnitudes (i.e., numbers) rather than continuous magnitudes (e.g., density, length, duration, etc.) (e.g., Cantlon et al. 2009; Gallistel and Gelman 2000). Much of humans’ higher quantitative abilities, including our symbolic number system, are grounded on numerical cognition and our intuitive “number sense” (Gallistel and Gelman 2005).

For a long time, quantitative cognition, and in particular numerical cognition, has been viewed as uniquely human and closely linked to language and education (Descartes and Lafleur 1960; Kant 1781; Ross 1908). During the last decades, however, comparative research has convincingly demonstrated that humans' closest living relatives, the nonhuman primates, possess basic quantitative abilities that can handle both continuous and discrete quantities. For example, primates are capable of absolute and relative quantity judgments (e.g., Beran 2004; Hanus and Call 2007; Hicks 1956; Jordan and Brannon 2006), they possess counting-like abilities (e.g., Beran 2001; Boysen and Berntson 1989; see Beran 2017 for a review) and even have the capacity to perform basic arithmetic operations (e.g., Beran 2001; Cantlon and Brannon 2007a; Flombaum et al. 2005). Together, these studies demonstrated that basic, non-symbolic numerical and other quantitative competencies exist independently of language or any formal training. Furthermore, the presence of quantitative abilities in a wide range of species highlights their ecological relevance in diverse contexts, such as foraging, spatial orientation, territorial selection and defense, or partner search.

The relative importance of quantitative information is further highlighted by the fact that humans spontaneously and automatically represent numerical information and even have difficulties inhibiting a response based on numerical stimulus dimensions (e.g., Dehaene 1997; Dehaene et al. 1993; Lefevre et al. 1988; Piazza et al. 2004). For nonhuman animals, by contrast, many researchers have argued that numerical representation is invoked only as a "last resort" strategy (e.g., Breukelaar and Dalrymple-Alford 1998; Davis 1993; Davis and Memmott 1982; Davis and Pérusse 1988; Seron and Pesenti 2001). That is, animals represent the numerical value of a stimulus only when there are no other relevant dimensions, such as shape or color, on which decisions can be based. This view was challenged when a comparative study found that rhesus monkeys' (*Macaca mulatta*) performance in a matching-to-sample paradigm was influenced by numerical ratio, even when number was pitted against other stimulus dimensions such as color or shape (Cantlon and Brannon 2007b). Hence, monkeys seem to represent numerical values naturally, and these numerical values appear to be of intrinsic relevance to them.

In sum, comparative research suggests that the last common ancestor of humans and other primates possessed basic quantitative abilities and may have used quantities and numbers spontaneously in various contexts.

Intriguingly, recent research shows that quantitative abilities may have evolved several times, leading to analogous capabilities in very distantly related species with highly distinct neuronal resources. Of particular relevance is a steadily growing body of research that has been dedicated to investigating quantitative abilities in invertebrates, which obtained some remarkable findings (see Bortot et al. 2020; Gatto et al. 2021; Pahl et al. 2013; Skorupski et al. 2018 for reviews). In one of the first studies, Chittka and Geiger (1995) showed that honeybees (*Apis mellifera*) remembered the number of prominent landmarks to relocate a food source, independently of the total flight distance. Later studies found that honeybees can be trained to discriminate exact numerosities, as long as the number of items within each set does not exceed four (Dacke and Srinivasan 2008; Gross et al. 2009) – the same upper limit that has also been suggested for primates (Barner et al. 2008; Hauser et al. 2000; Murofushi 1997; Tomonaga and Matsuzawa 2002). Honeybees were even found to spontaneously use quantities to discriminate food patches of differing quality (Howard et al. 2020b). Further, honeybees seem capable of basic arithmetic operations, such as addition and subtraction (Howard et al. 2019a). They can learn the association between numerosity and symbol (Howard et al. 2019b), which has been interpreted as a possible precursor ability of symbolic number representation (but see Howard et al. 2020a; Shaki and Fischer 2020). Another recent study (Howard et al. 2018) demonstrated that honeybees can learn "less than" and "more than" concepts, and even seemed to grasp a "concept of zero", i.e., an understanding that zero is a quantity at the low end of the positive numerical continuum (but see Shaki and Fischer 2020 for an alternative explanation for bees’ behavior). This capacity had previously only been demonstrated in primates and an African grey parrot (*Psittacus erithacus*) (Biro and Matsuzawa 2001; Pepperberg and Gordon 2005; Ramirez-Cardenas et al. 2016).

While shared quantitative abilities in humans and other primates can be traced back to similar neural mechanisms for quantity estimation (Dehaene et al. 1998), quantitative cognition findings in insects are particularly astonishing in light of the highly distinct neuronal capacities of insects' miniature brains. In an attempt to understand how quantity judgments can be achieved with such limited neuronal resources, neural network models have been created. This approach revealed that basic quantitative abilities, as reported in the studies mentioned above, can be achieved by a simple neural network consisting of only four independent neural units (Vasas and Chittka 2019) or, according to a recent study, with only a single spiking neuron (Rapp et al. 2020). When mimicking a behavioral task in free-flying bees that required quantitative cognition, the single spiking neuron model reached a similar success rate as found in honeybee experiments. Indeed, MaBouDi et al. (2020) found empirical support for the hypothesis that small-brained animals have developed different strategies to process quantity information than large-brained animals. In their study, honeybees did not determine the number of items in a set by using a rapid number assessment (as mammals would do); instead, they relied on sequential enumeration even when items were presented simultaneously and in small quantities. This process is more time-consuming than the rapid subitizing process mammals use, but it enables accurate enumeration of items despite the lower parallel processing capacity of small brains. Together, these findings suggest that there should be no limitation by neuronal capacity for possessing quantitative cognition, i.e., organisms with even smaller brains than honeybees might be capable of basic quantitative cognition.

Support for this prediction comes from quantitative cognition studies on a different family within the order of Hymenoptera: ants. Desert ants, *Cataglyphis*, were shown to measure distance by integrating step count (Wittlinger et al. 2006, 2007). *Formica xerophila* workers seem to assess quantitative factors such as their relative group size when deciding how to behave in competitive situations (Tanner 2006). More flexible usage of quantitative information was shown by d'Ettorre et al. (2021), who found that carpenter ants (*Camponotus aethiops*) spontaneously discriminated between differently sized piles of dummy cocoons. Additionally, they could be trained to identify a certain landmark by using its relative position within a sequence of landmarks (e.g., the third of five landmarks; also see Cammaerts & Cammaerts 2019a, b, c; Cammaerts & Cammaerts 2019a, b for experimental studies on quantitative cognition in *Myrmica sabuleti)*. Hence, research with ants suggests that insects with even fewer neuronal resources than honeybees are capable of flexible quantitative cognition. It remains an open question if also within the clade of bees, species smaller than honeybees, and thus with smaller brains, possess quantitative abilities.

Indeed, while there is a relative wealth of studies on honeybee quantitative cognition, other groups of bees have been neglected in this regard. To our knowledge, the only two studies investigating quantitative abilities in other bees tested a bumblebee (*Bombus terrestris*; Bar-Shai et al. 2011a) and a solitary bee (*Eucera spec*.; Bar-Shai et al. 2011b). Nothing is known about quantitative abilities in the largest tribe of eusocial bees, the stingless bees.

Stingless bees are a diverse monophyletic taxon, including some 60 genera with more than 500 species, and exhibit a pantropical and pansubtropical distribution (Rasmussen and Cameron 2010). Many stingless bee species forage throughout the year and some exhibit flower consistency as generalist pollinators (Slaa et al. 2006), indicating the potential to act as essential pollinators of economic plants (Amano et al. 2000; Bispo dos Santos et al. 2009; Heard 1988, 1994; Kakutani et al. 1993). Similar to honeybees, quantitative abilities could play a role for stingless bees in foraging contexts, e.g., to discriminate a larger patch of flowers from a smaller patch and keep track of numbers of landmarks to relocate a food source or the hive. Stingless bees are typically small, ranging between the size of a vinegar fly and a honeybee, and live in tropical and subtropical landscapes (Roubik 1992). Hence, there may be physiological (e.g., reduced brain size) or ecological features (e.g., foraging in a complexly structured environment) that might require different cognitive abilities in stingless bees compared to honeybees and bumblebees. So far, however, research has revealed commonalities in basic cognitive skills: Just like honeybees, stingless bees can also be trained to discriminate different colors (Sánchez and Vandame 2012; Spaethe et al. 2014), patterns (Sánchez and Vandame 2012; Moreno et al. 2012), and odors (Mc Cabe et al. 2007).

This study aimed to explore quantitative abilities in a small-brained species of stingless bees, *Trigona fuscipennis,* and investigate the importance of quantitative information relative to other stimulus dimensions. More specifically, we investigated whether stingless bees can discriminate two quantities (one and four) and whether quantitative information is a salient feature relative to three other essential flower attributes: color, shape, and surface area. Studying quantitative abilities in stingless bees will give us essential insights into the cognitive and neuronal prerequisites of quantitative cognition, including potential brain size limitations. It will also shed light on the ecological pressures that may have led to the emergence of quantitative and numerical abilities. Moreover, while previous studies have demonstrated that honeybees *can* use quantitative information, it remains unclear whether such information indeed plays a crucial role for bees in general compared to other stimulus dimensions. Considering that bees rely on nectar and pollen and need to be able to detect and discriminate rewarding flowers to collect their nutritional requirements, it is conceivable that color, shape, and perhaps surface area may be the most important sources of information for them. Quantitative information may play a crucial role for orientation, though, as it enables an individual to keep track of the number of landmarks in order to relocate a food source (Chittka and Geiger 1995). Hence, this study will provide us with essential information about the relative importance of quantitative information for pollinators. Lastly, this is the first study investigating quantitative abilities in a stingless bee, and, as such, it will add to our understanding of this highly important yet vastly understudied group of bees. Despite the fact that stingless bees are the largest group of eusocial bees and function as essential pollinators in the tropics, there is still a lack of studies exploring the cognitive abilities of these bees in comparison to the relatively well-studied honeybee.

## Methods

### Study site and species

The study was conducted in the Pacific southwest lowlands of Costa Rica, in the ecologically managed model farm "Finca Modelo" of the Tropical Research Station La Gamba, located at the border of the Piedras Blancas national park. The station is surrounded by primary and secondary rainforest. The experiments took place between February and March 2019, at the end of the dry season.

**Fig. 1:**
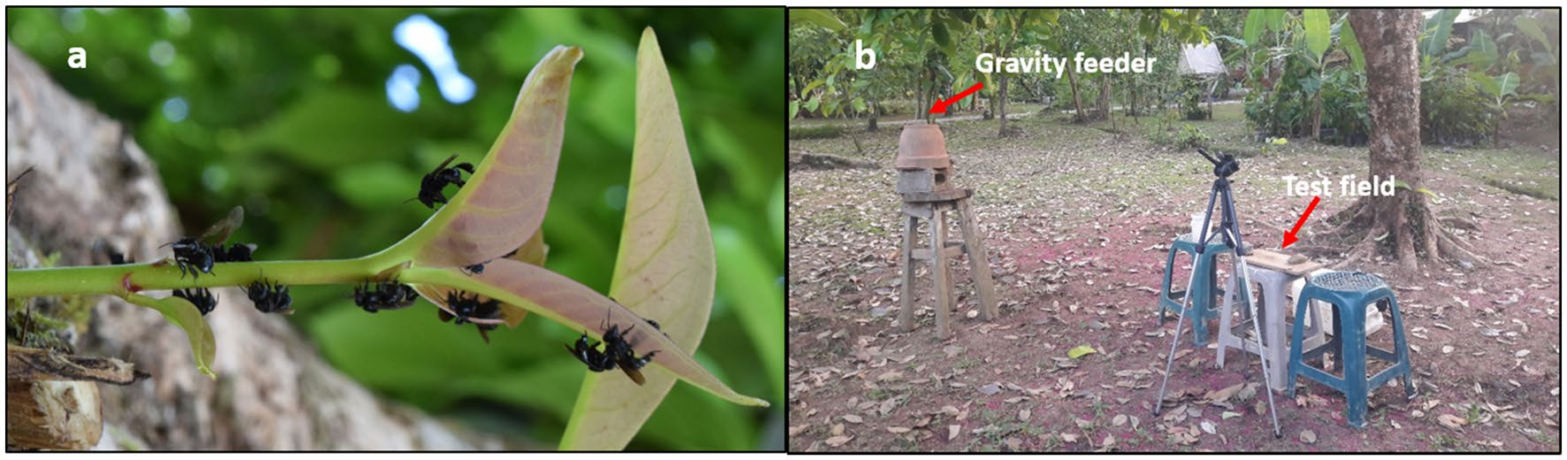
Study species and setup. **a** Workers of *T. fuscipennis* foraging in the "Finca Modelo" of the Tropical Research Station La Gamba. **b** Setup including the gravity feeder and the test field, on which stimuli were presented to individually marked forager bees

In all experiments, we tested free-flying foragers of *Trigona fuscipennis* (see Fig. 1a). *T. fuscipennis is* a species of stingless bees belonging to the New World Clade of the Meliponini (Rasmussen and Cameron 2010), which is common throughout Central America, as well as in Colombia and western Ecuador. Workers are relatively small (ca. 1.41 ± 0.04 mm intertegulae span; mean ± SD, *n* = 5; Spaethe et al. 2014) and usually forage in groups (Johnson and Hubbell 1975). The nests are commonly built in cavities of tree trunks or within termite nests. The entrances are characterized by funnel- or ear-shaped openings made of resin (Jarau and Barth 2008).

Foragers of *T. fuscipennis* were found to forage at a flowering bean plant in the Finca regularly. From these flowers, bees were trained about 2 m away to a gravity feeder at 0.75 m height offering 0.2 M sucrose solution. Subsequently, we trained individual bees (the subjects for the respective experiment) to visit the test field (see Fig. 1b). They were individually marked with a color code following a standard procedure used in previous experiments (Spaethe et al. 2014; Sommerlandt et al. 2014). In total, 215 individually marked bees participated in the study.

### Material

**Fig. 2:**
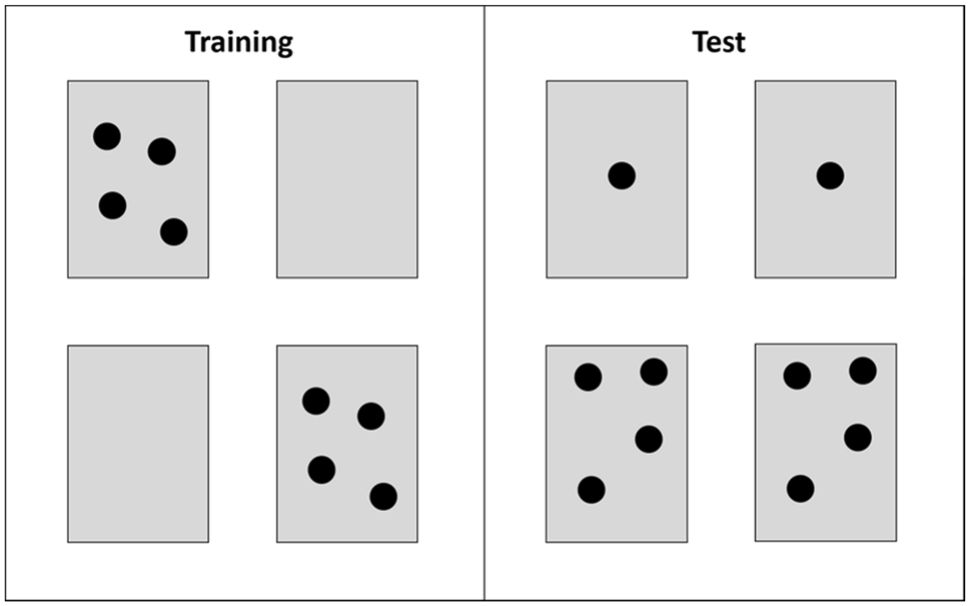
Example configurations of stimuli presented in the training and test of the *quantity only* experiment

The test field consisted of a blank gray laminated DIN A4 sized paper presented horizontally on a small table (height: 45 cm). On this test field, we presented the stimuli, which were laminated gray rectangles (6 cm × 4.2 cm) depicting either none, one, or four elements of different shapes, colors, and surface areas (see Fig. 2 for examples), depending on both experiment and condition.

### Design and procedure

Prior to each experiment, we trained naïve bees to visit the test field. Four different experiments were administered. Each of them consisted of a training and a test phase. During the training phase, bees were presented with four stimuli: Two identical samples (for around half of the bees, those were always cards depicting one element; for the other half, they depicted four elements) and two blank stimuli. The four stimuli were arranged in a 2 × 2 square with each stimulus' position randomized (see Fig. 2). The two samples were provided with a 10-µl drop of a 0.5 M sucrose solution as a reward, and the plain stimuli were supplied with the same amount of water. The position of the stimuli within the 2 × 2 square was randomly changed every 10 min to avoid position learning. Additionally, every 20 min, we replaced the cards with new stimuli (depicting different spatial configurations of the same number of elements). In total, training lasted 80 min, divided into eight blocks, 10 min each. Only bees that visited the test field in at least four of these training blocks were included in the subsequent test.

In the test, we again presented the bees with four stimuli: two identical cards depicting four elements and two identical cards showing one element. Importantly, bees in the test were confronted with a conflicting situation; While one stimulus type matched the sample with respect to the number of elements, but not with respect to one of several other stimulus dimensions (depending on the experiment, either shape, color, or surface area), the other stimulus type matched the sample in terms of this other stimulus dimension, but not in the number of elements (see Fig. 2). During testing, all stimuli were provided with a drop of water. The position of each stimulus within the 2 × 2 square was counterbalanced and changed every five minutes. After 10 minutes, a new set of the same stimulus (but with a different spatial arrangement of elements) were presented. After 20 min of test we conducted another 10 minutes of rewarded training (procedure as described above) to maintain the bees' motivation throughout the otherwise non-rewarded test. Subsequently, the test was continued as described for a further 20 min. Hence, the test consisted of eight 5-minute blocks. For each bee, we recorded all choices (i.e., touches or landings on the stimuli) during the test phase.

#### Quantity only

**Fig. 3:**
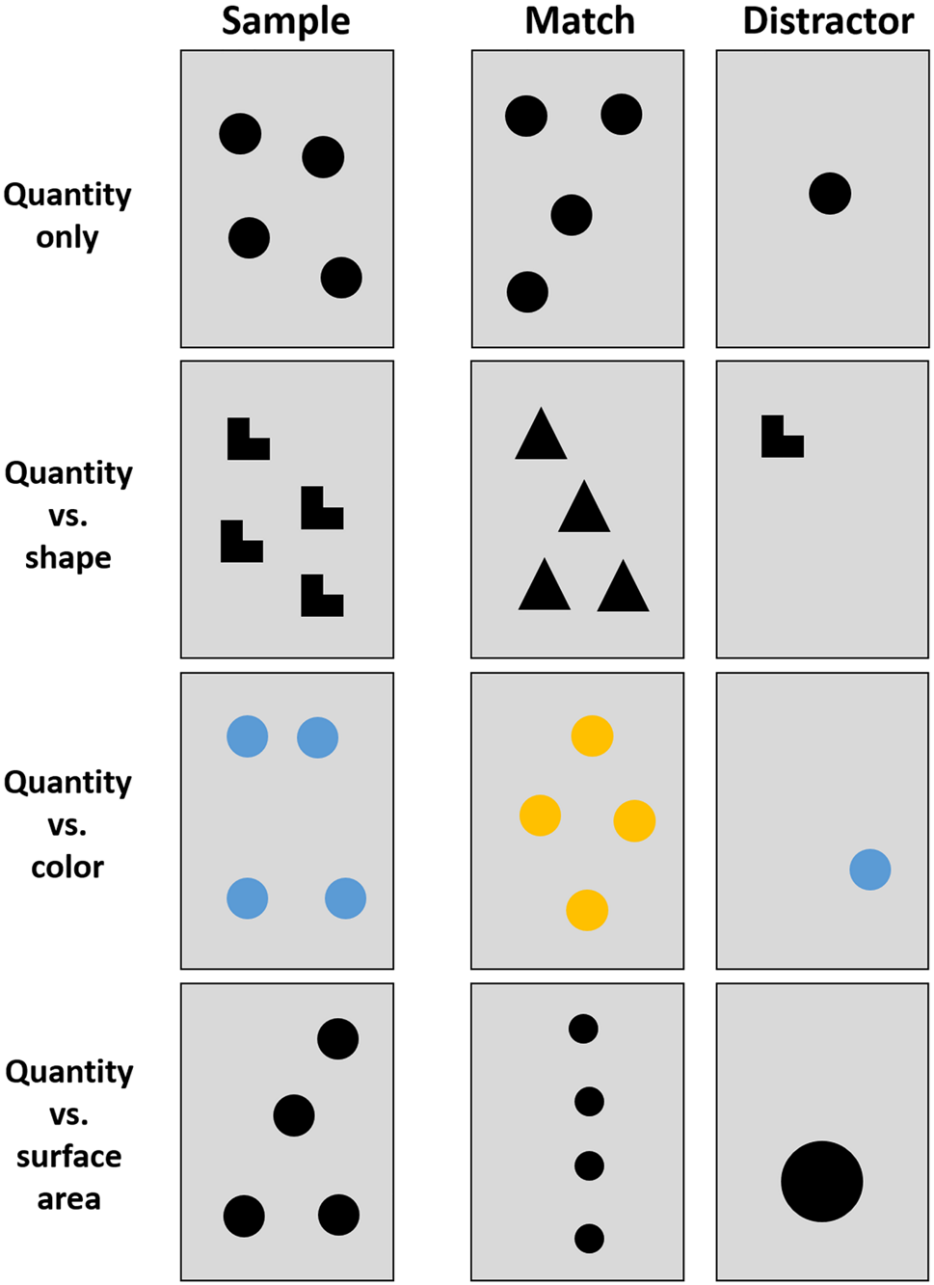
Examples for stimulus combinations in all four experiments

In this experiment, we aimed to investigate whether stingless bees are, in principle, able to learn and discriminate different quantities of elements. Therefore, 49 individual bees were trained to discriminate between one and four elements (*N* = 24 were rewarded on the single-element stimulus and *N* = 25 on the four-element stimulus; see Fig. 3).

#### Quantity versus shape

In this experiment, we examined whether bees, when associating a stimulus with a food reward, memorized the stimulus' quantitative features, i.e., the number of elements depicted on it, or instead learned the shape of the individual elements. We trained a total of 78 bees on stimuli depicting either a single element (*N* = 41; for 29 of those, the trained elements were triangles) or four elements (*N* = 37; for 26 of those, the trained elements were triangles). In the subsequent test, bees were given a choice between stimuli that matched the sample either in quantity or in shape (see Fig. 3).

#### Quantity versus color

The aim of this experiment was to explore whether bees pay attention to a stimulus' quantitative features or rather to its color. We trained a total of 48 bees on stimuli depicting either one element (*N* = 18; for 8 of those, the trained elements were blue) or four elements (*N* = 30; for 11 of those, the trained elements were blue). A preference test conducted with other individuals from the same colony revealed that the bees had no strong bias for either of these two colors (see supplementary information SI Fig. 1). In the subsequent test, bees were given a choice between stimuli that matched the sample either in quantity or in color (see Fig. 3).

#### Quantity^1^ versus surface area

In this experiment, we examined whether bees memorized a stimulus' quantitative features, i.e., the number of elements depicted on it, or instead learned the cumulative surface area of the elements. We trained a total of 45 bees on stimuli depicting either a single element (*N* = 17) or four elements (*N* = 28). In the test, the numerical match elements had either a 50% smaller cumulative surface area than in training (*N* = 27, see Fig. 3) or a two times larger cumulative surface area (*N* = 18). The stimuli not matching the sample in terms of number, by contrast, had the same cumulative surface area of elements as the training stimulus (see Fig. 3).

### Statistical analysis

Data and analysis code are freely available in an online repository (https://github.com/manuelbohn/bees). All analyses were computed in R version 4.1.0 (R Core Team 2021). We analyzed the data in three steps. First, we aggregated the data for each individual in each experiment and compared the proportion of choices based on quantity to a level expected by chance (50%). We used one-sample t-tests for this analysis.

Second, for each experiment, we ran a generalized linear mixed model (GLMM) with a logit link on the trial-by-trial data. The purpose of this analysis was to test if the quantity the subject was trained with or other features of the stimuli influenced choice behavior. All models included the quantity the individual was trained on as a fixed effect, subject as a random intercept and trial (within block) and block as random slopes. For *quantity vs. shape*, we included the shape of the training stimuli as an additional fixed effect. For *quantity vs. color*, we included the color of the training stimuli and for *quantity vs. surface*, we included the size of the test stimuli (relative to the training stimuli) as a fixed effect. Additional interaction terms could be included in the models. However, because we had no a-priori theoretical expectation about such interactions, we did not include them in the main analysis. For completeness, we include them in the associated analysis file in the online repository. Models were fit using the function glmer from the package lme4 (Bates et al. 2015). P-values for predictor variables were calculated based on likelihood ratio tests (LRT) computed via the function drop1.

Finally, we compared performance across experiments. We fit a single GLMM to the trial-by-trial data with experiment and the quantity individuals were trained on as fixed effects. The random effects were the same as in the by-experiment models. Again, we evaluated the importance of the fixed effects using LRTs. In addition, we computed pairwise contrasts (with adjusted *p*-values) using the function emmeans from the package emmeans (Russel 2021).

## Results

**Fig. 4:**
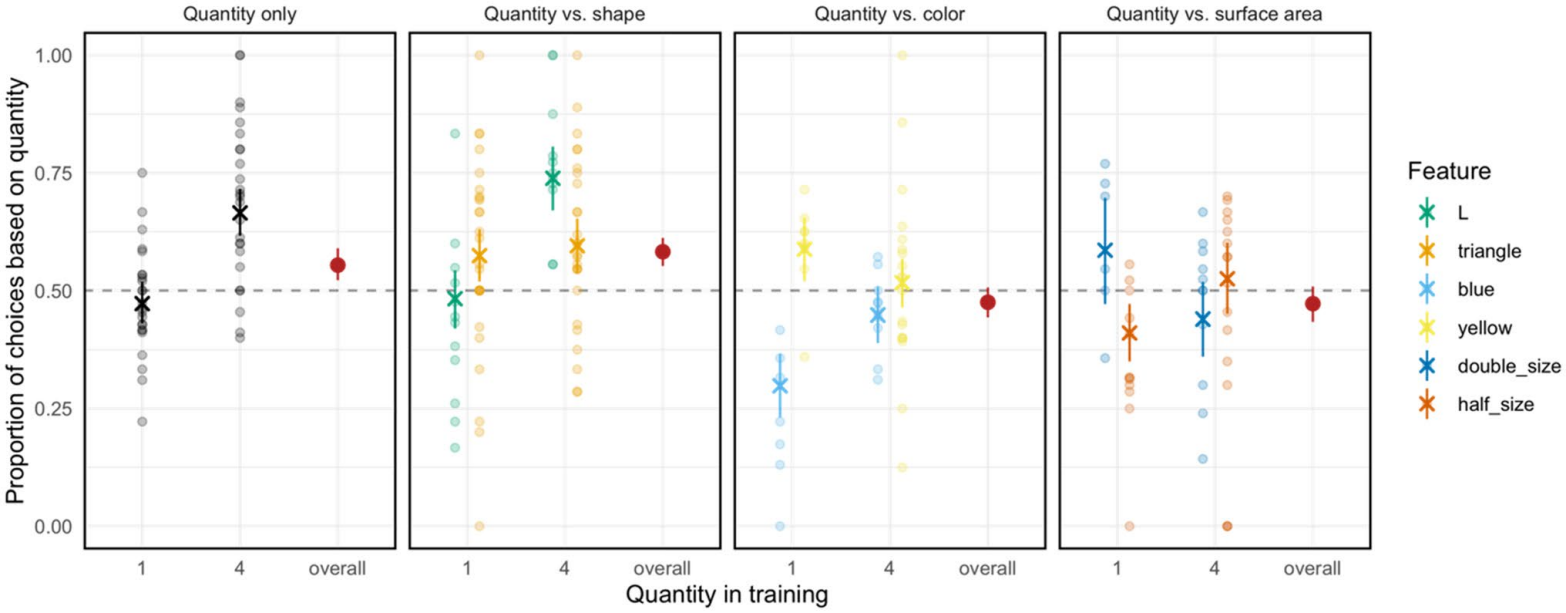
Proportion of choices based on quantity in the four experiments. Red dots in each facet show the mean (with 95% confidence interval (CI) based on non-parametric bootstrap) for the respective experiment. Colored crosses give the mean (with 95% CI) when sub-setting the data based on training sample quantity and/or feature. Light dots show individual datapoints

Figure 4 visualizes the results. Bees made choices based on quantity at a rate above chance in *quantity only* (*m* = 0.59, sd = 0.18, *t*(48) = 3.37, *p* = 0.001, *d* = 0.48) and in *quantity vs. shape* (*m* = 0.59, sd = 0.21, *t*(75) = 3.37, *p* < 0.001, *d* = 0.42). Choices were not different from chance in the other two experiments.

In the analysis focusing on each experiment separately, we found the following: In *quantity only*, bees were more likely to make a choice based on quantity if they were trained on four elements (*β* = 0.80, se = 0.16, *Χ*^2^(1) = 22.97, *p* < 0.001), but performance was around chance when trained on one element (see Fig. 4). We found a similar pattern in *quantity vs. shape* (*β* = 0.49, se = 0.17, *Χ*^2^(1) = 7.82, *p* = 0.005) while the shape of the sample had no effect on bees’ choice behavior. In *quantity vs. color*, we did not see an effect of training quantity, but one of sample color (*β* = 0.49, se = 0.17, *Χ*^2^(1) = 7.82, *p* = 0.005). Bees made choices based on quantity below chance when trained with blue stimuli and at a higher rate (but not above chance) when trained with yellow stimuli. For *quantity vs. surface*, neither the number of elements nor the size of the test stimuli influenced bees' choices.

When combining the data from all experiments, we found an overall effect of sample quantity—bees were more likely to make a choice based on quantity when trained with four elements (*β* = 0.42, se = 0.09, *Χ*^2^(1) = 21.16, *p* < 0.001). We also found that choice behavior differed between experiments (*Χ*^2^(1) = 25.28, *p* < 0.001). Pairwise comparisons indicated that quantity-based choices were higher in *quantity only* compared to *quantity vs. color* (Odds ratio (OR) = 1.15, se = 0.20, *p* = 0.007) and *quantity vs. surface* (OR = 1.47, se = 0.20, *p* = 0.031) but not to *quantity vs. shape* (*p* = 0.833). Quantity choices were also more frequent in *quantity vs. shape* compared to *quantity vs. color* (Odds ratio (OR) = 1.69, se = 0.21, *p* < 0.001) and *quantity vs. surface* (Odds ratio (OR) = 1.62, se = 0.21, *p* = 0.001). Finally, we found no difference between *quantity vs. color* and *quantity vs. surface* (*p* = 0.992).

## Discussion

### Quantity only

The aim of this experiment was to investigate if stingless bees can be trained to differentiate two quantities (one and four) from each other. Overall, we found that bees chose correctly (i.e., they chose their trained quantity in the test) significantly more often than what would have been expected by chance. This suggests that (i) bees did form some association between stimuli and reward during the training, and (ii) they could discriminate between different quantities of elements, namely between one and four. These results resemble findings of studies on honeybees (e.g., Chittka and Geiger 1995; Gross et al. 2009; Howard et al. 2018) and suggest that stingless bees, too, are capable of basic quantitative cognition.

A less expected finding was that performance was strongly influenced by the quantity the bees were trained on: While performance was above chance-level when they had been trained on four elements, their performance was not different from chance when they had been trained on one element. Previous studies on honeybees, by contrast, showed that bees could be trained to discriminate both larger from smaller and smaller from larger quantities (Gross et al. 2009; Howard et al. 2018). A likely explanation for the stingless bees' behavior may be found in our methodology, which was slightly different from prior honeybee studies: In most of the previous studies, subjects were trained differentially. That is, in training, they learned to choose a certain quantity of elements over other quantities (in the case of absolute quantity discrimination; e.g., Gross et al. 2009) or to choose the larger/smaller of two quantities (in the case of relative quantity discrimination; e.g., Howard et al. 2018). Then, they were required to find this specific quantity (or the larger/smaller of two) with new stimulus examples in the test. By contrast, in the present study, bees were trained with a single quantity against blank stimuli (gray cards) and were only introduced to a second quantity in the test phase. (This training design was necessary to contrast quantity information with other stimulus dimensions in the subsequent experiments). Hence, unlike previous studies, stingless bees in our study did not experience that a quantity-related strategy would lead to success during training. Bees' failure to choose the previously rewarded quantity "one" over the newly introduced "four" is most likely due to a trade-off between two intuitive strategies. One strategy is based on the configuration of elements on the stimuli and implies approaching those stimuli that appear most similar to the training stimuli (i.e., stimuli depicting one element). The other strategy is based on the individual element(s) of the stimuli. It implies approaching those stimuli, which have *more* of the previously rewarded elements (i.e., stimuli depicting four elements) in a "more of something good is always better" fashion. Both strategies separately can be sensible to optimize foraging, and, in the case of the bees trained on "four," lead to the same, above-chance-level results (because the stimulus most similar to the training stimulus was also the one containing more elements). For bees trained on "one," however, the two strategies lead to contrasting choices, and hence, when combined/torn between them, lead to chance-level performance.

Support for this explanation comes from a recent study on honeybees' spontaneous quantity discrimination: In a training phase, Howard et al. (2020b) rewarded bees on stimuli depicting a single element. In the test, this single element was paired with stimuli depicting other quantities. The authors reported that bees spontaneously preferred the higher quantity in comparisons that involved, e.g., one versus four elements. These results suggest that, when non-differentially trained, honeybees, too, tend to apply the "more of something good is always better" foraging rule. Notably, the training in Howard et al.'s study involved only rewarded stimuli depicting one element, whereas our training always included non-rewarded plain stimuli. Hence, in contrast to the honeybees, stingless bees in our study were given the additional information that they need to pay attention to the presence or absence of elements on the stimuli. This may have primed the "approach stimuli which appear most similar to the training stimuli" rule. This methodological difference may account for the clear choice for more in honeybees, and the chance-level choice of stingless bees, since only the latter were likely undecided between two possible strategies.

In contrast to the bees in our study, primates can discriminate quantities up to a certain ratio threshold with very high levels of accuracy, even when trained to select the smaller of two quantities (e.g., Cantlon and Brannon 2007b). Interestingly, however, in specific situations, primates also have difficulties overcoming the "more of something good is always better" heuristic: When discriminating between quantities of food, both apes (Boysen and Berntson 1995) and monkeys (Schmitt and Fischer 2011) failed to select the smaller quantity in order to receive the larger reward. However, if the stimuli consisted of inedible objects instead of food items, the subjects' performance in the same task was significantly enhanced (Schmitt & Fischer 2011). Given that stingless bees in the present study formed a strong association between the elements on the stimuli and a food reward in training, perhaps they had similar difficulties inhibiting the intuitive tendency to select the larger of the two quantities of elements in the test.

An alternative explanation for bees’ chance level performance when trained on one element is peak shift. Peak shift is a common phenomenon in discrimination learning and has also been demonstrated in honeybees and bumblebees (see, e.g., Andrew et al. 2014; Leonard et al. 2011; Lynn et al. 2005; Wright et al. 2008, 2009). It describes the phenomenon that, if animals are trained with two similar stimuli such that one is rewarding and one punishing, then, following training, animals show a greatest preference not for the rewarded stimulus, but for a novel stimulus that is slightly more different from the punishing one. In the current study, when bees had to discriminate the stimulus depicting one or four elements from a blank stimulus during training, this may have induced a peak of responding beyond the trained quantity, further away from the “zero” stimulus. That is, peak shift may have biased bees to choose a greater quantity of elements than they were trained on. This may explain why bees trained on one element did not select this trained quantity in the test more often than would have been expected by chance. However, peak shift is most prominent when both the trained (rewarded and unrewarded) stimuli, as well as the novel stimuli are very similar. In our case, since the novel “four elements” are relatively far from the trained “one element,” the bias for the larger quantity induced by peak shift was likely weak, resulting in roughly equal choices between the two options for the bees trained on one element. For the bees trained on four elements, peak shift could not have biased choices beyond the conditioned stimulus because there was no larger available quantity in the test. We cannot determine whether bees’ performance patterns in the current study are due to a “more is better” rule or due to peak shift. However, if peak shift played a role for bees in the context of quantity discrimination, one would expect specific performance patterns if one tested bees with several different quantities. More specifically, bees trained on one vs. zero elements should show a clear preference for two elements when confronted with one vs. two elements in the test because two is only slightly more different from zero (as unrewarded quantity) than one. This preference for the larger quantity should be less clear if those bees were confronted with one vs. three elements in the test.

In sum, while the current study demonstrated that stingless bees could be trained to discriminate quantities, it remains unclear whether stingless bees' abilities reach a similar extent as has been reported for honeybees. Future studies using differential conditioning techniques should investigate whether "priming" bees during the training to pick the smaller of two quantities and testing them with new examples of the same quantities leads to improved performance. Additionally, future studies should test bees with several different quantities to investigate if peak shift plays a role in the context of quantity discrimination.

### Quantity vs. shape

The aim of this experiment was to examine whether stingless bees pay more attention to the number of elements depicted on a stimulus or to the shape of these elements. We found that the bees' choice behavior closely matched their behavior in the *quantity only* experiment: Overall, bees chose based on quantity significantly more often than what would have been expected by chance. Shape, by contrast, did not have an influence on the bees' choices. Interestingly, just as in the *quantity only* experiment, bees trained on four elements performed better than bees that were trained on one element.

The finding that bees behaved as if they had only been confronted with quantitative information and ignored shape information demonstrates that, for these stingless bees, quantity trumps shape as a salient stimulus dimension. From an ecological perspective, these results seem reasonable: As flying foragers, bees approach flowers from various angles, resulting in variable appearances of their shape, which renders shape a somewhat unreliable cue for flower quality. The quantity of flowers, by contrast, is a very reliable cue for the amount of food available in a patch and therefore seems to be a salient stimulus. This explanation is supported by a study on a different species of stingless bees, *Scaptotrigona mexicana* (Sánchez and Vandame 2012). In this study, it was found that foragers of *S. mexicana* can be trained to discriminate different stimulus shapes. Still, they performed notably less well than in a color discrimination task, suggesting that shape plays only a minor role for stingless bees in foraging situations.

Findings comparable to those obtained in the current experiment were obtained in a study on rhesus monkeys (Cantlon and Brannon 2007b). In this study, monkeys spontaneously matched a sample based on the number of elements rather than their shape, at least when the numerical ratio between match and distractor was large enough. Hence, quantitative information seems to trump shape information in several distantly related species, emphasizing the crucial role of quantity for decision-making throughout evolutionary history.

### Quantity vs. color

This experiment aimed to investigate whether stingless bees pay more attention to the number of elements depicted on a stimulus or to the color of these elements. Overall, bees did not choose differently than what would have been expected by chance, i.e., bees did not base their decisions on quantitative information. Also, bees made fewer quantity-based choices compared to both the *quantity only* experiment and the *quantity vs. shape* experiment. Instead, their choice was strongly influenced by color. Specifically, bees were more likely to make quantity-based choices when they were trained on yellow stimuli (whose numerical match was blue) than when they were trained on blue stimuli (whose numerical match was yellow). In other words, bees tended to choose blue stimuli over yellow ones, regardless of their depicted quantity. This is surprising because a non-rewarded preference test conducted with different individuals of the same group revealed no strong preference for one of the two colors over the other. Previous research has demonstrated that color preferences within the same species of honeybees or bumblebees may vary between studies (e.g., Balamurali et al. 2018; Giurfa et al. 1995; Raine and Chittka 2007). This is probably due to the fact that the tested individuals likely had different prior experiences with different colors, independently of the studies. Assuming that color preferences are shaped by individual experience, it is thus conceivable that they may change not only between studies and populations, but also between individuals of the same population, and likely even within individuals across time points.

Interestingly, while the color of their training stimuli influenced bees' choice behavior, bees did not approach their trained color in the test more often than expected by chance (this would have been indicated by quantity-based choices below chance-level in our analysis). Instead, their preference for blue over yellow masked any learned association between either quantity and reward or color and reward. Such strong color preferences show that color is arguably among the most salient cues for stingless bees as pollinators, most likely because color reliably indicates both the location and quality of a flower as a source of food.

However, previous studies suggested that stingless bees can overcome color preferences with extensive training. For instance, Sánchez and Vandame (2012) successfully trained foragers of *Scaptotrigona mexicana* to associate a wide range of different colors with rewards and to discriminate these trained colors from other colors. Moreover, foragers of *Trigona fuscipennis* can differentiate different colors (blue, yellow, pink) from gray tones (Spaethe et al. 2014). Still, to date, it remains unclear whether, and with how much training, this species can be trained to overcome their color preferences. Either way, the current study suggests that for stingless bees, color information trumps quantitative information. Intriguingly, a comparable study on rhesus macaques (Cantlon and Brannon 2007b) showed that this might also be the case for nonhuman primates: When monkeys were required to match a sample either based on the number of its elements or their color, only those monkeys that had previously been trained to discriminate quantities matched the sample based on the number of elements. Number-naïve monkeys, by contrast, matched the sample based on its color. The saliency of color information was previously also demonstrated in research with birds (Kazemi et al. 2014). In an avoidance-leanring task, blue tits learned to avoid different colors, shapes, and patterns. Among these features, color information was learned at the highest rate, implying that it had the highest saliency for the birds. Consequently, it is conceivable that color is a very salient stimulus feature for many different animal species, perhaps exceeding the saliency of quantity.

### Quantity vs. surface area

This experiment aimed to examine whether stingless bees would intuitively match a stimulus based on the number of its elements or rather on their cumulative surface area. We found that bees' choice did not seem to be influenced by either of the two factors. Instead, bees performed at chance-level throughout the experiment. While these findings appear surprising at first glance, a straightforward explanation could be that both factors' influence on bees' decision was equally strong, therefore evening out their respective effect. By contrast, a similar study on rhesus monkeys (Cantlon and Brannon 2007b) found that even number-naïve monkeys intuitively based their choice on the number of elements on a stimulus, disregarding their cumulative surface area.

Future studies should investigate whether stingless bees can be trained to discriminate between different cumulative surface areas. If so, they should replicate our experiment using several different quantities, and systematically vary the ratio between the two quantities (e.g., 1 vs. 5; 1 vs. 3; 1 vs. 2). The presence of a ratio effect on performance (i.e., individuals choose based on quantity more often when confronted with 1 vs. 5 compared to 1 vs. 2) would indicate an influence of numerical quantity even in the absence of an "over-chance" performance. This would allow for a much more fine-grained analysis of the seemingly competing influences of quantitative information and surface area information than was possible in the current study.

### General discussion

The aim of this study was, first, to investigate if free-flying stingless bees can be trained to differentiate two quantities (one and four) and, second, to explore the importance of quantitative information relative to other stimulus features. We found that workers of *T. fuscipennis* can discriminate one from four elements, thus providing the first hint that stingless bees, like honeybees, possess basic quantitative competencies. This finding is particularly interesting in light of the limited neuronal resources of these small-brained eusocial bees. Thus, it supports the idea that basic quantitative abilities can be achieved with simple neural networks (MaBouDi et al. 2020; Vasas and Chittka 2019) or even single specialized neurons (Rapp et al. 2020). An important limitation of this study was that we could only contrast two quantities (one and four). While this finding is an essential first step in exploring the quantitative abilities of stingless bees, future research should expand on these findings and test bees' abilities in discriminating several different quantities. In honeybees, just as in most vertebrate species, performance in quantity discrimination tasks is ratio-dependent (e.g., Howard et al. 2019b; Bortot et al. 2020). That is, honeybees perform better when the magnitude of difference between the quantities to be discriminated is relatively large (e.g., 1 vs. 4) compared to when it is very small (e.g., 1 vs. 2). It will be an intriguing question for future research to investigate if also stingless bees show a ratio-dependent performance, and whether the “signature limit”, i.e. the smallest ratio that can still be discriminated, is comparable between stingless bees and honeybees, despite their difference in brain size. Interestingly, we found that stingless bees did select the larger of the two quantities when they were rewarded on this quantity in training. They did not do the same with the smaller of the two quantities, though. Most likely, this curious finding is due to a trade-off between two contrasting strategies to solve the task: one aiming to choose the stimulus that is most similar to the training stimulus (the match), and the other seeking to optimize the outcome by selecting the stimulus containing more of the previously rewarded elements (the distractor). Prospective studies should further explore this hypothesis by using a differential conditioning task setup to investigate whether bees can be trained to select the smaller of two quantities. Alternatively, the observed performance patterns could be due to peak shift processes induced by the discrimination learning paradigm. To shed more light on the role of peak shift in the context of quantity discrimination, future research should test bees with several different quantities and investigate how performance changes if the distractor stimulus gets closer to the unrewarded training stimulus.

While the results of the present study suggest basic quantitative abilities in stingless bees, it remains an open question if these abilities are grounded on representations of discrete quantity (i.e., numbers) or on representations of continuous quantity (i.e., non-numerical magnitudes). In all our tasks, numerical quantity was confounded with at least one continuous dimension (e.g., density or convex hull, see, e.g. Leibovich et al. 2017). This resembles virtually every naturally occurring situation, where numerical and non-numerical quantitative information is typically strongly correlated. For instance, among several trees of the same species, the one with the most flowers will usually also have the largest area covered by flowers and the highest density of flowers. Also, it will most likely be the one with the highest intensity of smell. Therefore, it is advantageous for animals to possess quantitative abilities that can deal with all kinds of magnitudes and flexibly select the most relevant one. Indeed, research suggests that vertrebrate animals are able to combine representations of different kinds of (continuous and discrete) magnitudes (Walsh 2003). Strictly numerical abilities can only be revealed if all non-numerical features are controlled, which is an inherently different task. For several species, including honeybees, previous research has found evidence suggestive of numerical cognition (see e.g., Gatto et al 2021; Pahl et al. 2013; but see Leibovich et al. 2017). It will be an interesting question for future research to disentangle if stingless bees possess numerical cognition and if they readily rely on discrete rather than continuous quantities in their decisions.

Interestingly, our findings show that quantity is a salient feature for foraging stingless bees, apparently trumping shape information. This result speaks against the hypothesis that quantitative representation is invoked in animals only as a "last resort" strategy (Breukelaar and Dalrymple-Alford 1998; Davis 1993; Davis and Memmott 1982; Davis and Pérusse 1988; Seron and Pesenti 2001). Instead, it shows that quantitative information processing is of high relevance even in small-brained insects. The finding that bees seem to attend more to quantity than to shape is ecologically valid since a flower’s shape might not be a reliable cue for its quality. In contrast, the number of flowers might well be a reliable cue for the amount of food available in a patch. The most salient cue for stingless bees, however, seems to be the color of a stimulus. Strong preferences for specific colors masked the bees' performance in the current associative learning task, suggesting that color preferences guide stingless bees' foraging activities to the most profitable flowers. It is important to note that in the present study, we only tested one pair of colors, shapes, and surface areas, respectively. Thus, it is possible that bees would perform differently with other pairings within each stimulus dimension. Future research should look at each stimulus dimension more thoroughly and contrast several different versions against each other to shed more light on this possibility.

Our study suggests both commonalities and differences in the relative importance of various stimulus attributes for bees and primates. For rhesus monkeys, Cantlon and Brannon (2007b) suggested that numerical quantity is among the most salient stimulus features, followed by color. For stingless bees, quantity and color are salient stimulus dimensions as well. However, bees in our study seemed to be guided mainly by color preferences rather than newly formed associations with colors (whereas monkeys more flexibly associated different colors with rewards). In contrast to the monkeys, for stingless bees also surface area seemed to be of relevance. One could speculate that this difference may be due to differences in diets and foraging styles. For bees as pollinators, surface area might be more salient than for primates because the surface area of a flower can indicate its flowering stage (if the flower is still closed, its surface area is smaller than when it is fully open). Hence, bees might be guided in their foraging decisions by the color of a flower (indicative of species and nectar quality), the number of flowers in a patch (indicative of the amount of nectar per area), and their surface area (indicative of the flowering stage and nectar availability). Rhesus macaques, as a frugivorous species, might instead mostly base their foraging decisions on both the number of available fruits in a given spot (indicative of the available amount of food) and their color (indicative of the fruits’ ripeness and edibility). The surface area covered by those fruits, by contrast, seems to be less informative for these primates. To shed more light on species differences concerning the relative salience of different stimulus dimensions, we must test more species in paradigms similar to the one we implemented here. It will be an intriguing avenue for future research to further explore the potential influence of foraging style and diet on the relative importance of stimulus features across species.

In sum, the current study is the first to demonstrate that a species of stingless bees possesses basic quantitative abilities and preferentially uses quantitative information (besides color information) to locate food resources. These findings add to our overall understanding of the evolution of quantitative cognition and contribute to a better understanding of this vastly understudied group of essential tropical pollinators.

## Supplementary Information

Below is the link to the electronic supplementary material.

Supplementary file1 (PDF 386 KB)

## Data Availability

Data is freely available in an online repository (https://github.com/manuelbohn/bees).

## Footnotes

1 Please note that, in this case, the term quantity refers to all (continuous and discrete) quantitative features except surface area.

## Code availability

The analysis code is freely available in an online repository (https://github.com/manuelbohn/bees).
