# Supplementary material for: Does quantity matter to a stingless bee?
Source: Anim Cogn. 2021 Nov 23;25(3):617–29. doi: 10.1007/s10071-021-01581-6 (PMC9107420; doi:10.1007/s10071-021-01581-6)
Supplement: Supplementary file 1 — Supplementary file1 (PDF 386 KB) [file 10071_2021_1581_MOESM1_ESM.pdf]

Does quantity matter to a stingless bee?

Johanna Eckert*[1,2,], Manuel Bohn[1], Johannes Spaethe[2]

[1]Department of Comparative Cultural Psychology, Max Planck Institute for Evolutionary Anthropology, Leipzig, Germany.

[2]Department of Behavioral Physiology and Sociobiology, University of Wuerzburg, Germany

*Corresponding author: johanna_eckert@eva.mpg.de
Department of Comparative Cultural Psychology, Max Planck Institute for Evolutionary Anthropology, Deutscher Platz 6, 04103 Leipzig, Germany.
ORCID: 0000-0001-8161-082

Supplementary information

Preference test color blue vs. yellow

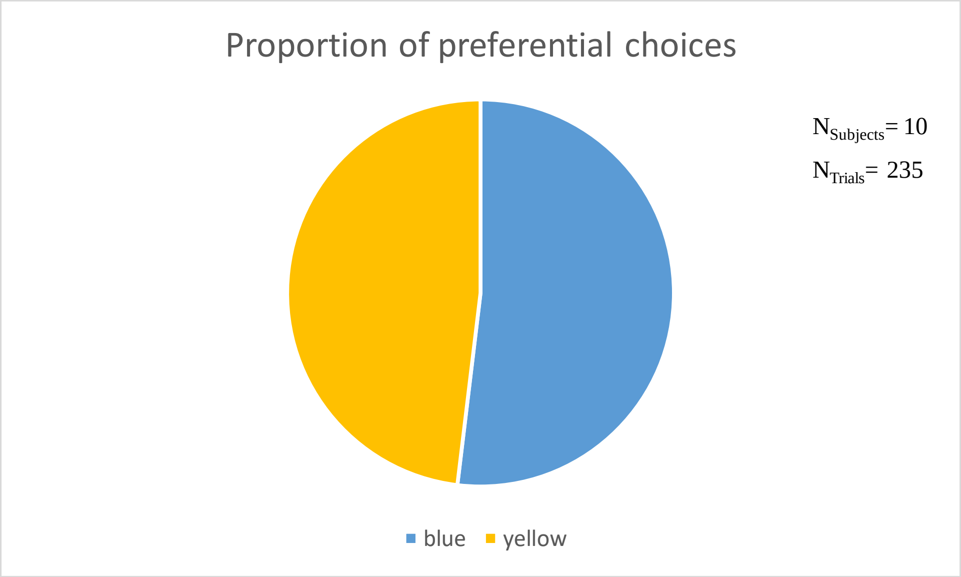

SI Fig 1 Results of the color preference test. Ten bees were tested in a non-rewarded preference test and given the choice between stimuli containing a blue or a yellow element. In 122 out of 235 trials, bees chose the blue stimulus. In 113 out of 235 trials, they chose the yellow stimulus.
